# Polymer chemistry: fundamentals and applications

**DOI:** 10.3762/bjoc.17.200

**Published:** 2021-12-14

**Authors:** Bernhard V K J Schmidt

**Affiliations:** 1 School of Chemistry, University of Glasgow, G12 8QQ Glasgow, UK

**Keywords:** polymer chemistry

Ever since the introduction of the term macromolecule and the early days of polymer science [1], organic chemistry and polymer chemistry have been closely related [2]. It only seems to be a logical step to give room for a thematic issue on polymer chemistry in the *Beilstein Journal of Organic Chemistry*. The connection between organic and polymer chemistry has been highlighted frequently [3–4], up to the point where one can talk about macroorganic chemistry*,* where oligomers bring molecular precision from organic chemistry together with materials properties from polymer chemistry [5]. Especially in the challenge of transformation to a more sustainable polymer science, organic chemistry can give significant support in the development of greener polymer materials [6].

This thematic issue covers a broad range of current topics in polymer chemistry, from synthesis to materials and applications. In the area of synthetic methods, the use of click photochemistry in polymer and organic molecule synthesis is presented, as well as the combination of polymers with supramolecular chemistry for the assembly of polymer fibers and the synthesis of well-defined polysaccharides. In the direction of materials, cryogels and reinforced hydrogels are discussed, as well as 3D-printed poly(caprolactone) biomaterials. In addition, properties of thermoresponsive materials and self-healing materials are presented in this thematic issue. Finally, the use of polymerization-induced self-assembly for the synthesis of drug carriers is presented. The issue also shows the multidisciplinary character of polymer science, for example when supramolecular motifs, organic coupling reactions or photocatalysis are employed in the development of new polymer materials in order to address specific applications.

Overall, I hope the thematic issue “Polymer chemistry: fundamentals and applications” highlights the breadth of current polymer research and enables the reader to dive into this fascinating area of chemistry. I want to express my gratitude to all authors who showcase their excellent work in this thematic issue and the Editorial Team for the magnificent organization.

Bernhard V. K. J. Schmidt

Glasgow, November 2021
